# CAR Immunotherapy for the treatment of infectious diseases: a systematic review

**DOI:** 10.3389/fimmu.2024.1289303

**Published:** 2024-01-30

**Authors:** Elena Morte-Romea, Cecilia Pesini, Galadriel Pellejero-Sagastizábal, Santiago Letona-Giménez, Luis Martínez-Lostao, Silvia Loscos Aranda, Carla Toyas, Sergio Redrado, Elena Dolader-Ballesteros, Maykel Arias, Eva M. Galvez, Rebeca Sanz-Pamplona, Julián Pardo, Jose Ramón Paño-Pardo, Ariel Ramírez-Labrada

**Affiliations:** ^1^ Infectious Diseases Department, Hospital Clínico Universitario Lozano Blesa, Zaragoza, Spain; ^2^ Fundación Instituto de Investigación Sanitaria Aragón (IIS Aragón), Biomedical Research Centre of Aragon (CIBA), Zaragoza, Spain; ^3^ Centro de Investigación Biomédica en Red en Enfermedades Infecciosas, Instituto de Salud Carlos III (CIBERINFEC), Madrid, Spain; ^4^ Immunotherapy, Cytotoxicity, Inflammation and Cancer, Aragón Health Research Institute (IIS Aragón), Biomedical Research Centre of Aragón (CIBA), Zaragoza, Spain; ^5^ Department of Immunology, Hospital Clínico Universitario Lozano Blesa, Zaragoza, Spain; ^6^ Department of Microbiology, Pediatry, Radiology and Public Health, University of Zaragoza, Zaragoza, Spain; ^7^ Nanoscience Institute of Aragon (INA), Consejo Superior de Investigaciones Científicas (CSIC), University of Zaragoza, Zaragoza, Spain; ^8^ Instituto de Carboquímica-Consejo Superior de Investigaciones Científicadas (ICB-CSIC), Zaragoza, Spain; ^9^ Centro de Investigación Biomédica en Red en Epidemiología y Salud Pública, Instituto de Salud Carlos III (CIBERESP), Madrid, Spain; ^10^ Unidad de Nanotoxicología e Inmunotoxicología Experimental (UNATI), Fundación Instituto de Investigación Sanitaria Aragón (IIS Aragón), Biomedical Research Centre of Aragón (CIBA), Zaragoza, Spain

**Keywords:** CAR cells, infectious diseases, CAR, immunotherapy, viral infections

## Abstract

Immunotherapy treatments aim to modulate the host’s immune response to either mitigate it in inflammatory/autoimmune disease or enhance it against infection or cancer. Among different immunotherapies reaching clinical application during the last years, chimeric antigen receptor (CAR) immunotherapy has emerged as an effective treatment for cancer where different CAR T cells have already been approved. Yet their use against infectious diseases is an area still relatively poorly explored, albeit with tremendous potential for research and clinical application. Infectious diseases represent a global health challenge, with the escalating threat of antimicrobial resistance underscoring the need for alternative therapeutic approaches. This review aims to systematically evaluate the current applications of CAR immunotherapy in infectious diseases and discuss its potential for future applications. Notably, CAR cell therapies, initially developed for cancer treatment, are gaining recognition as potential remedies for infectious diseases. The review sheds light on significant progress in CAR T cell therapy directed at viral and opportunistic fungal infections.

## A basic introduction to immunity and immunotherapy

A healthy host immune system eliminates pathogens and tumor cells while preserves tissue homeostasis, beneficial commensal flora, food, or environmental factors such as harmless materials derived from nature or man-made (1). Immunotherapy encompasses various treatments designed to modulate the immune response. It can dampen the immune response in inflammatory or autoimmune diseases or boost it to combat infections or cancer. William B. Coley, an orthopedic surgeon of the late 19th century, is now regarded as the father of immunotherapy. He observed that patients who underwent surgery for bone sarcomas and developed infections in the surgery sites exhibited spontaneous regression of the non-resected tumor. Then, he injected inactivated *Streptococcus pyogenes* into sarcoma sites, observing tumor shrinkage. Since then, the accumulated knowledge of the mechanisms regulating the immune response has led to the development of different ways to safely and effectively modulate its action to treat disease (2).

Cells from innate immunity, like macrophages, mast cells, or granulocytes, are the main mechanisms to eliminate extracellular pathogens in concerted action with B cell-derived antibodies. Meanwhile, cytotoxic T (Tc) and Natural Killer (NK) cells are crucial for eliminating cancer cells and intracellular pathogens using cytotoxic molecules like death ligands (i.e., FasL or TRAIL) and perforin and granzymes (3). All these responses are activated by a finely regulated inflammatory response involving different types of cytokines like interleukins (IL), or chemokines (CCL, CXCL) and cells like dendritic cells or monocytes (4). In addition to eliminating intracellular pathogens and cancer cells, recent evidence indicates that Tc and NK cells are also active participants in controlling extracellular infections, including bacteria and fungi. Once the pathogen is eliminated and immune homeostasis is recovered, long-lived pathogen-specific memory T and B cells are generated. Memory T/B cells mediate a faster and more potent response against future infections with the same pathogen.

Similarly to memory T/B cells, some cells from innate immunity, like macrophages or NK cells are able to respond faster to a new infection. In this case, this response is not pathogen-specific and might help to control infections caused by other pathogens. This process, known as trained immunity, is emerging as a novel therapeutic alternative with wide implications in vaccination for infectious diseases and cancer immunotherapy (5).

Although both Tc and NK cells cooperate in controlling pathogens and cancer and share cytotoxic mechanisms, their response against offending cells is different. Activation of CD8^+^ T cells and generation of Tc cells requires the recognition of pathogen-derived peptides presented by Major Histocompatibility Complex-I (MHC-I) molecules through the T Cell Receptor (TCR) together with co-stimulatory signals provided by specific receptors and cytokines like IL2 and IL12. MHC-I and co-stimulatory signals are provided by professional antigen-presenting cells (APC), mainly dendritic cells (DC). Meanwhile, cytokines are generated by CD4^+^Th1 cells that have been previously differentiated from naïve CD4^+^T cells through the interaction of TCR with pathogen-derived peptides presented by MHC-II in DCs together with co-stimulatory molecules and Th1-polarizing cytokines like IL12 (5). In contrast to Tc cells, NK cell activation is antigen-independent and regulated by a complex integration of positive and negative signals originated from NK cell activating and inhibitory receptors after recognizing their corresponding ligands in target cells (6).

Additionally, negative regulatory feedback controls Tc and NK cell activation, preventing cell hyperactivation and preserving self-tolerance. This mechanism is known as immune checkpoints (ICs). Some of them are proteins expressed on the Tc and NK cells membrane shortly after they get activated that exert inhibitory functions modulating the activation process. Among the most studied inhibitory ICs are Programmed Cell Death 1 (PD-1) and Cytotoxic T lymphocyte-associated Protein 4 (CTLA-4) (7). The binding of these molecules with their ligands (PD-L1 and CD80/CD86, respectively) inhibits T and NK cell functions and might be used by tumours and pathogens to avoid their elimination (8).

The increasing knowledge of the mechanisms described above has allowed modulating aberrant immune responses to treat disease and, thus, developing more effective and safer immunotherapies. The approved treatments include vaccines, monoclonal antibodies, cytokines, immunomodulatory antibodies (i.e., antiPD1 or antiCTLA4), and, more recently, adoptive T cell therapy (ACT), mainly CART cells (9). However, the development of immunotherapy treatments showing high efficacy has been mainly pushed by the oncology field. Still, as indicated above, the immune response against infection is similar to the immune response against tumors. And the immunosuppressive microenvironment found in chronic difficult to treat infections is also comparable to that promoted by cancer cells, including a high expression of ICs (10). Thus, immunotherapy treatments effective in cancer might be applied to treating some infectious diseases, especially those resistant to current treatments due to the increasing global challenge of multidrug-resistant bacteria.

Additionally, the difficulties in developing new antimicrobial agents and the increased relevance of chronic viral infections in immunocompromised patients have shifted scientific and medical attention to immunotherapy following the excellent results in oncology. In particular, ACT, and more specifically, CAR cell-based therapies, has positioned itself as a promising opportunity for treating infections with few therapeutic alternatives. Here, we will systematically review the main results available from preclinical and clinical studies that support this potential, focusing on CART cells. However, a brief introduction to CAR cells will be presented before that.

## Principles of CAR cell design and production

**Figure 1:**
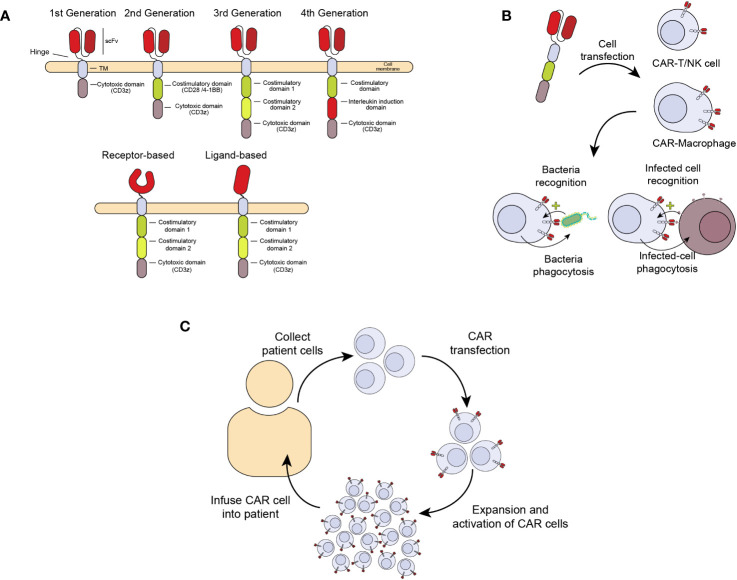
Generation of chimeric antigen receptors (CAR) expressed in cells from the immune system with antimicrobial activity. **(A)** Chimeric receptor involves an antibody fragment (scFv) or receptor against an antigen followed by a hinge peptide, a transmembrane domain, and one or more intracellular domains of proteins like the CD3 zeta (ζ) chain of the T-cell receptor complex (CD3ζ) and CD28 or 4-1BB (CD137), with the ability to signal and induce proliferation, activation, and cytotoxicity in the cell expressing the CAR. **(B)** Once the extracellular scFv/receptor domain of the CAR recognizes the antigen in an infected cell, the CAR cell gets activated and eliminates the infected cells. **(C)** CAR cell production. The manufacturing process begins with collecting peripheral blood mononuclear cells from a patient by leukapheresis. The cells are enriched, selected, and activated, followed by transduction with a self-inactivating lentiviral vector containing the CAR transgene. Cells are expanded following transduction until the final product dose requirements are met. Once the patient is ready, the cells are infused into the same patient who provided the leukapheresed cells.

A relatively new and promising ACT treatment is the generation of chimeric antigen receptors (CAR) expressed in cells from the immune system with cytotoxic and anti-infectious potential, including T (CAR-T) and NK (CAR-NK) cells and macrophages (CAR-M) (11). CARs are chimeric receptors consisting of a targeting element, usually an antibody fragment (scFv, single chain fragment variable) or a receptor against the antigen of interest. This sequence is followed by a hinge peptide, a transmembrane domain, and one or more intracellular signaling domains, like those from the CD3 zeta chain of the T-cell receptor complex (CD3ζ) or the coestimulatory molecules CD28 or 4-1BB (CD137). These domains confer the ability to induce proliferation, activation, and cytotoxicity of CAR-expressing cells (**Figure 1A**) (12). In this way, when the extracellular scFv/receptor domain of the CAR recognizes an antigen, the CAR cell gets activated and eliminates the pathogen or infected cell (**Figure 1B**). **Figure 1C** summarizes the process of CAR cell production for patient treatment. This therapy has already been approved for haematological cancers like refractory acute lymphoblastic leukemia, lymphoma and multiple myeloma (13). However, prior to this clinical success, the CAR development has undergone several improvements to enhance their persistence and effectiveness. The first CARs that were designed consisted of only one activation domain (First-generation CARs), which made them very prone to anergy and persisted for a short time *in vivo*, reducing their efficacy. With the development of second-generation CARs, including a co-stimulatory receptor, a great improvement in activation and proliferation was achieved, showing high efficacy *in vivo*, which was key for clinical approval (14). Although more sophisticated CARs have been developed, including several co-stimulatory signals and other molecules like cytokines or neutralizing antibodies, none have been approved for clinical use. Nowadays, the persistence of second-generation CAR-T cells in cancer patients may last many months to years (15, 16).

After this brief description of CAR cell design and functioning, we will systematically analyze the ongoing CAR cell-based approaches in viral, fungal, and bacterial infections and propose an infection-suitable model to develop and test novel CAR cell-based treatments.

## Methods

### Information sources and search strategy

We searched the PubMed, Scopus, Cochrane, databases from their inception to February 13, 2023 (**Supplementary Tables 1-3**). The following keywords were used to identify studies of interest: “chimeric antigen receptor T cell”, “CAR-T cell”, “chimeric antigen receptor NK cell”, “CAR-NK cell”, “chimeric antigen receptor macrophage cell”, “CAR-M cell” “Infectious Diseases”, “bacterial diseases”, “viral diseases”, “fungal diseases”, “bacterial infections”, “viral infections” and “fungal infections”. We combined the search terms differently, as shown in **Supplementary Tables 1-3**. The goal was to identify research papers and published clinical trials using CAR cells to treat infectious diseases.

### Study selection and data extraction

Two researchers independently identified and reviewed eligible full-text articles. Only English-language articles were included. The following data of interest were then extracted from the included studies: DOI, PMID, titles, publication year, first author, infections, pathogens, type of cell, methods for transduction, target molecules, receptor used in the CAR design, and the number of patients treated. Disagreements were resolved by careful discussion between the researchers.

## Results

### Study selection

**Figure 2:**
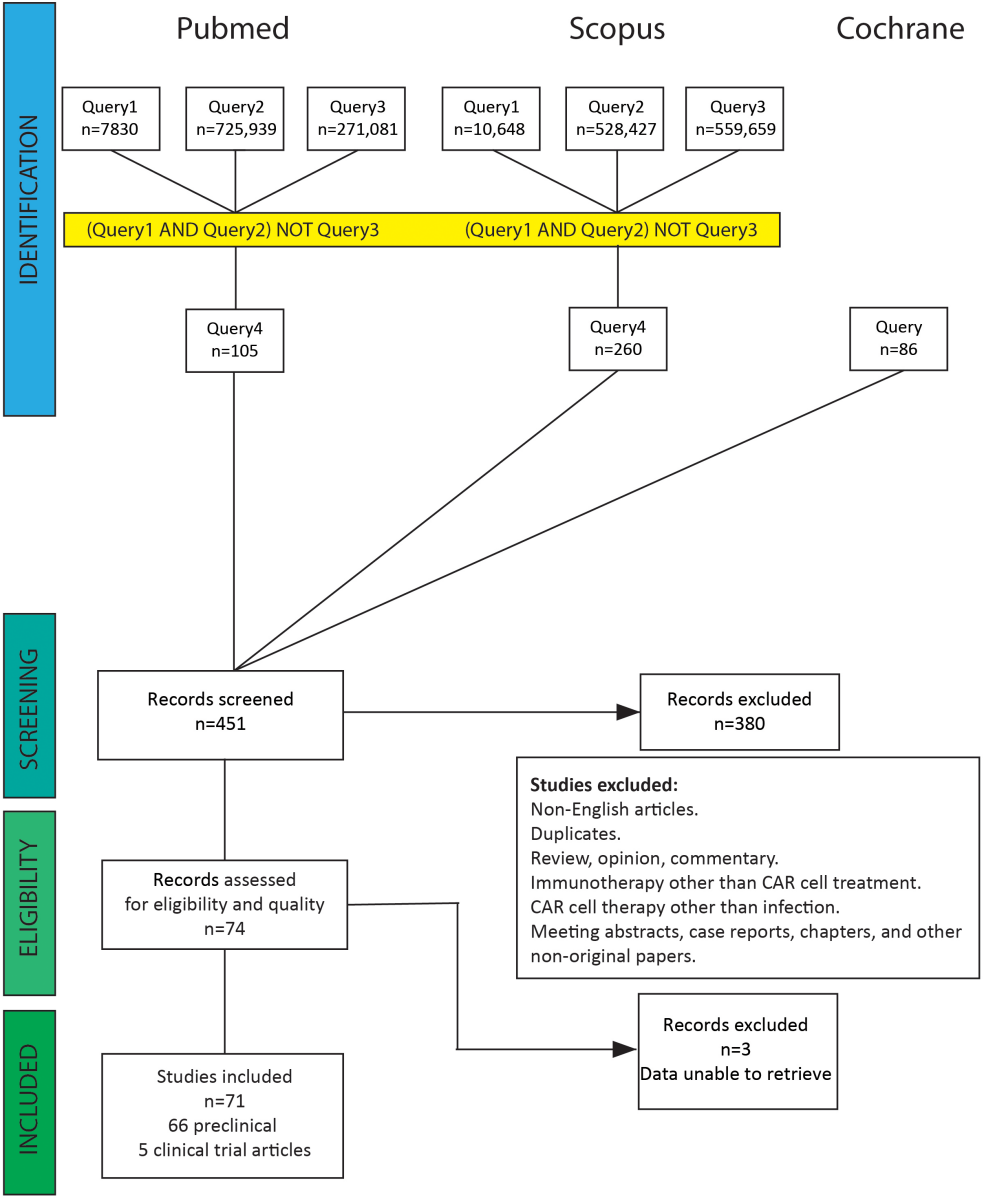
PRISMA flow diagram for literature search. A total of 451 abstracts were identified from the electronic database search. After removing non-English articles, duplicates, reviews, opinions, commentary, meeting abstracts, case reports, chapters, and CAR cell therapy other than treating infectious diseases, 74 abstracts were selected and reviewed. Three clinical trial studies were excluded because the results had not yet been published.

Publications were selected according to our search strategy. After screening by title and abstract, we discarded articles on tumor diseases, haematological malignancies, immunotherapy other than CAR cell treatment, duplicates, and non-English articles. Subsequently, studies covering the area of CAR cell treatment for infections were checked by reading titles and abstracts. After filtering out reviews, opinions, commentary, meeting abstracts, case reports, chapters, and other non-original papers, we collected 71 original studies of CAR cell therapy for infectious diseases (**Supplementary Table 4**). **Supplementary Tables 1-3** and **Figure 2** show the literature screening process. The literature search yielded eight clinical trials, of which three were excluded because the results had not yet been published. One publication was a long-term follow-up of patients from the first clinical trials. The remaining 66 articles were preclinical investigations published in peer-reviewed journals.

### Description of selected publications

Overall, five papers from clinical trials fulfilled the inclusion criteria. Trials were conducted from 2000 to 2021. Two papers refer to the same clinical trial. One was a phase I clinical trial, two were phase II clinical trials, and one did not specify. All of them studied CAR-T cell therapy against Human Immunodeficiency Virus (HIV) infected patients; the clinical trials included a total of 104 patients. Regarding CAR design, three of them were first-generation CARs containing the extracellular domain of human CD4, which targets the viral envelope glycoprotein-120 (gp-120) expressed on the surface of infected cells and the CD3ζ chain. The other trial used a 4^th^ generation CAR, consisting of a scFv against gp-120 linked to the CD3ζ chain and CD28 and 4-1BB (CD137) co-stimulatory domains, and a combination of sh-PD-1, sh-LAG-3, and sh-TIM-3. They all expressed the CAR on T cells by lentiviral or retroviral transduction methods. These studies were designed to mainly assess the safety and feasibility of the adoptive transfer of CAR-T cells into patients positive for HIV-1, although, as described in more detail below, some of them also analysed viral reservoir levels.

**Figure 3:**
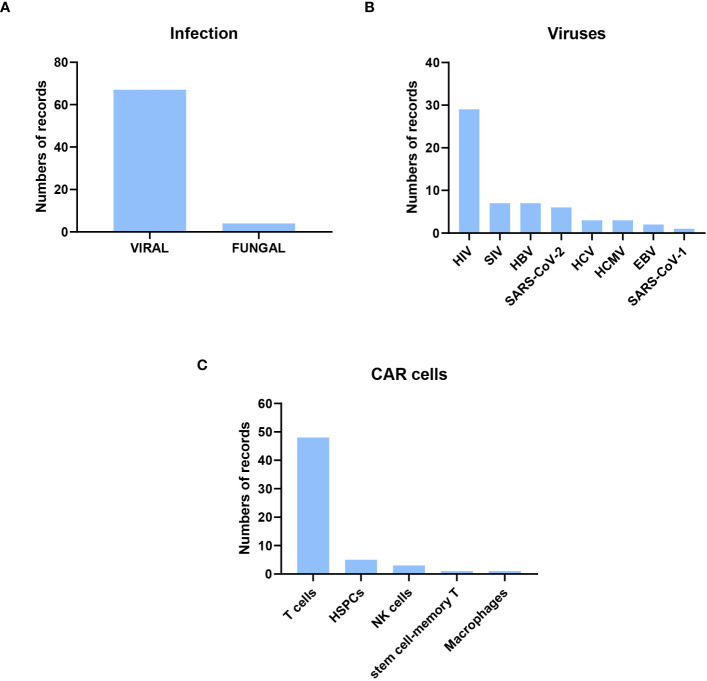
Description of the preclinical research published from their inception to February 13, 2023. Most of the original papers were about CAR cells to treat viral infection, focusing on HIV viruses and using CAR T cells. **(A)** Distribution of Infection Types: Out of the original papers, 67 focused on viral infections, while four centered on fungal infections. **(B)** Distribution of Viral Infections: The predominant CAR designs targeted HIV-infected cells. **(C)** Distribution of CAR-expressing Cell Types: T cells were the most commonly employed cells for expressing CAR constructs.

Of the preclinical research publications, 67 were on viral infections, only four on fungal infections, two on *Cryptococcus* spp, and two on *Aspergillus* spp. In the case of viral infections, the most frequent (50%) were on HIV, followed by Simian immunodeficiency *virus* (SIV) and hepatitis B virus (HBV). Other publications with CAR cells against other viral infections, such as SARS-CoV-1 and -2, were also found (**Figure 3**). The cells used to express CAR were mainly T cells and, to a lesser extent, NK cells, HSPCs, macrophages, and memory T cells. The methods used to transform the cells were lentiviral, retroviral, and gammaretroviral transduction. Only five publications used electroporation, always in T cells, as the election method to obtain CAR cells, and one of them used CRISPR. scFv was used in most CAR designs except for HIV, where the extracellular domain of human CD4 was more frequently used (**Supplementary Table 2**). Below, a more detailed analyses of the studies described is presented.

#### HIV infection

**Figure 4:**
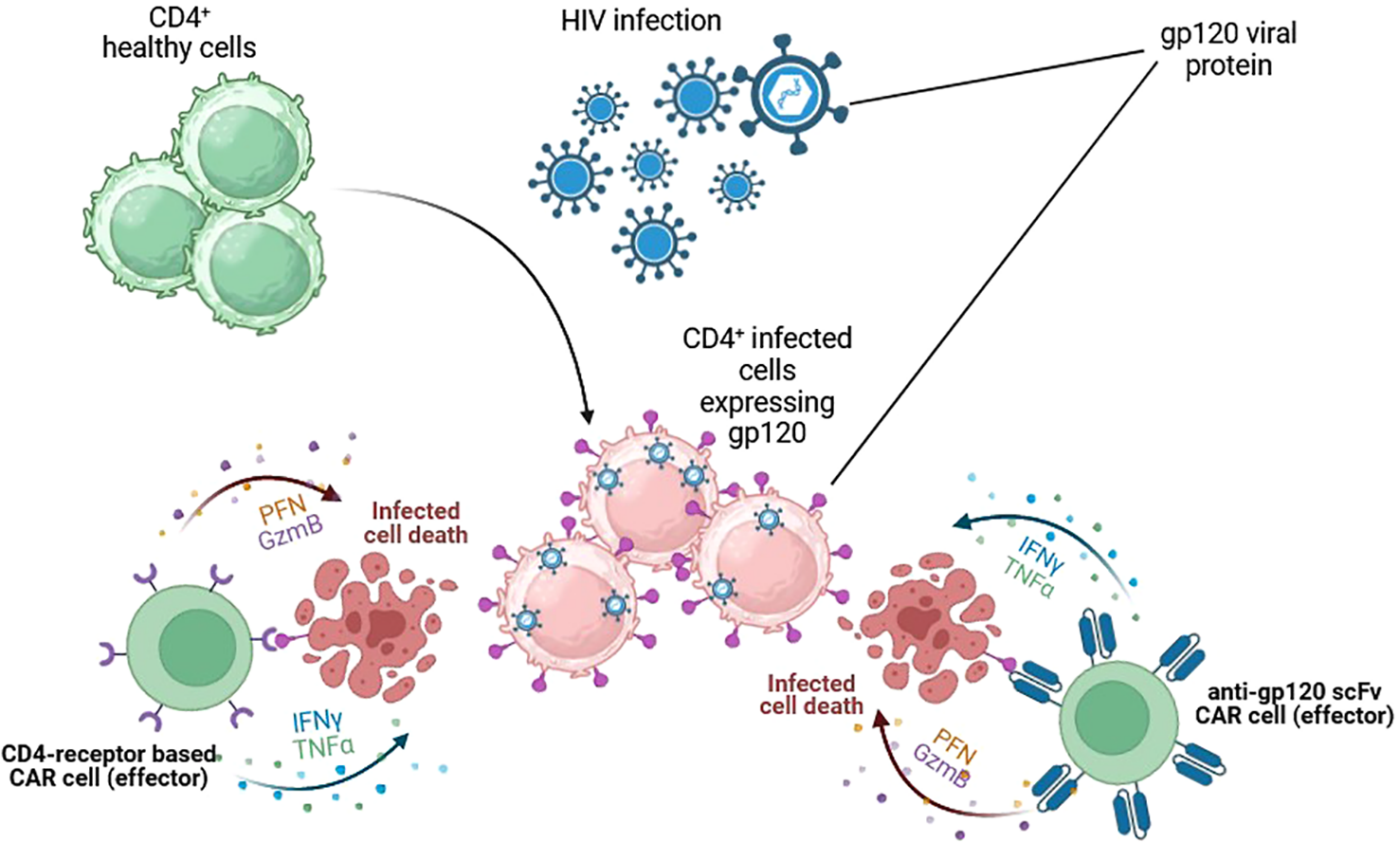
CAR cells targeting HIV-infected cells. Cytotoxic cells are redirected against HIV-infected cells by the expression of gp-120-specific CARs on their surface. Two main structures have been used. The extracellular domain of CD4 receptors and broadly neutralising scFv antibodies. Both target the gp-120 and could successfully kill HIV-infected cells and control HIV infection. Figures created using BioRender.com.

HIV was first discovered in 1984 due to atypical and fatal cases of pneumonia that typically affected men. Since then, millions of people have become infected in low- and high-income countries. HIV is a retrovirus transmitted through unprotected sexual contact, vertical transmission from mother to child, or parenteral transmission. It produces chronic immunosuppression by depleting CD4^+^ T lymphocytes, leading to opportunistic infections. HIV infection has become a frequent target for CAR-T therapy due to the requirement for a chronic, lifelong combination of antiretroviral therapy (ART) to achieve stable suppression of plasma viremia. HIV infects several target cells by using the viral envelope gp-120 protein, inducing different ways of immunosuppression, including CD4+T cell elimination and downregulation of MHC expression in infected cells (17), a mechanism that justified the interest in CAR-T development. CAR-T therapy for HIV infection was first described in 1990s in combination with ART. Despite promising initial data in preclinical experiments (18–20), subsequent clinical trials found minimal impact on virus persistence (21). Two main structures have been used to design anti-HIV CAR-T: CD4 receptors and broadly neutralising antibodies, which target the gp-120 and deactivate most circulating HIV strains (**Figure 4**) (22).

Between 2000 and 2002, three clinical studies evaluated the CD4ζ (extracellular domain of human CD4 linked to the zeta chain) CAR expressed in T cells in subjects with active viremia. In 2000, two clinical trials with CD4ζ-modified T cells from HIV-infected subjects (autologous cells) or from identical twins of HIV-infected subjects (syngeneic cells) were published with 30 and 24 patients, respectively (23, 24). In 2002, a phase II clinical trial was published in 40 patients with ART and plasma viral loads <50 copies/ml that were randomized or not to HIV-specific CD4+ and CD8+ CAR-T cells binding HIV gp-120. During the first 24 weeks after infusion, fewer viral rebounds (viral blips or relapses) were detected in the CAR-T arm, although this trend was not conserved over time (25).

These trials confirmed the safety and the stable persistence of gene-modified T cells in the blood. However, they did not find any significant difference in HIV load in blood or tissue reservoirs (25).

Using first-generation CAR-T cells lacking co-stimulatory domains might have limited the control of viral replication observed in these clinical trials. In 2021, another clinical trial was published with 14 patients (26) using a second-generation CAR consisting of a high-affinity scFv fused with a CD28 and 4-1BB co-stimulatory domains. The study’s primary objectives were to assess the safety and side-effect profile and evaluate the pharmacokinetic characteristics of a single dose of CAR-T cells. In addition, ART interruption was performed to investigate the antiviral effect of CAR-T cell therapy, showing that the CAR-T cells failed to inhibit viral propagation, likely due to preexisting or CAR-T cell treatment–driven, resistant viruses. This result suggested that CAR-T cells recognised the infected cells and exerted immune pressure, resulting in a selection of viruses with mutations in the antigens recognized by CAR-T cells (27). This issue could be overcome by using scFvs targeting various antigens on the viral envelope.

Other clinical trials in this line are ongoing or in the recruitment phase (28). Similarly to solid tumours, one of the main challenges of HIV CAR-T therapy is increasing the infiltration of CAR-T cells in HIV tissue reservoirs (lymph nodes, gut, spleen, or central nervous system) where few CAR-T cells might be detected (29).

#### Epstein-Barr virus

Immunotherapy could also provide novel strategies to treat other chronic viral infections, particularly those induced by herpesviruses in immunocompromised patients. This potential stems from the unique ability of this viral family to enter a state of true latency and subsequently reactivate. Epstein-Barr virus (EBV) is a human herpesvirus with a high seroprevalence. In immunocompetent patients, it causes a self-limiting systemic infection known as infectious mononucleosis. However, chronic infections due to EBV in immunocompromised patients might predispose them to more severe diseases, including malignant diseases like Burkitt’slymphomas and B-cell lymphoma and other malignancies like nasopharyngeal carcinomas and soft-tissue tumors.

The EBV latent membrane protein 1 (LMP1), an integral membrane protein key in viral replication, is one of the viral gene products essential for B-cell transformation (30). During latency, the virus may be partially or completely hidden from the immune system. The viral genome is present, but gene expression is limited, infectious virus is not produced, and, thus, the immune system can not recognize it (26). Most EBV-infected subjects control acute infections by cytotoxic immune cell responses via NK and T cells. Only a few infected individuals will develop chronic EBV-associated pathologies, often related to immune deficiencies (26). Here, CAR cell therapies might be an option to treat infection and avoid further complications.

The first CAR-T cell therapy against EBV chronic infection has been analysed *in vitro* and *in vivo* models in nasopharyngeal carcinoma cells. The results seem to indicate a protective response able to control infection and prevent virus-mediated carcinogenesis (31). This study used a second-generation CAR consisting of anti-LMP1 scFv and CD28-CD3ζ sequence expressed in T cells by lentiviral transduction. Other preclinical studies using anti-EBV CAR-T therapy have shown efficacy in controlling EBV-associated lymphoproliferative disorders using CAR cells targeting the EBV envelope glycoprotein 350 (gp-350). This protein is expressed abundantly during EBV lytic reactivation and sporadically on the surface of latently infected cells (32, 33). Several phase I and II clinical trials are ongoing or recruiting, most of which are targeted to LMP1 or other clusters of differentiation on the B cell surface (34).

#### Cytomegalovirus

Cytomegalovirus (CMV) is a highly prevalent pathogen that causes elevated morbidity and mortality in immunocompromised patients. It can induce organ-specific diseases like colitis, retinitis, and hepatitis, but it can also induce immunosuppression, leading to opportunistic infections. In addition, it has been related to acute and chronic transplant rejection, including graft versus host disease (GvHD) and allograft dysfunction. In order to prevent these serious complications, it is of utmost importance to prioritize the management of CMV viremia in individuals who have undergone solid organ transplantation (SOT) or hematopoietic stem cell transplantation (HSCT). This becomes particularly significant given the constraints associated with antiviral therapies such as ganciclovir and letermovir, whose efficacy is affected by the development of drug resistance and potential toxicity. As with other herpesvirus, CMV can evade T-cell responses by downregulating the expression of MHC (35).

Using ACT based on CMV-specific T cells isolated from healthy latently infected patients has undergone extensive research in HSCT patients (35, 36). The importance of this therapy is confirmed by the recommendation of the European Conference on Infections in Leukemia, prioritizing this option in patients with refractory CMV infection (37). However, this therapy is still limited by the availability of compatible T-cell donors and the lack of sufficient studies in SOT patients (38). Due to these limitations, different studies have analysed the potential efficacy of CAR-T cells in treating CMV infections. In *in vitro* and humanized *in vivo mouse* models, different CMV CAR-T cell therapies have shown encouraging results (39). The most relevant antigen to target CMV is glycoprotein B, abundantly expressed in the virus envelope and essential for virus entry and cell-to-cell spread (38). The first publication of a CMV-specific CAR consisted of a scFv against the glycoprotein B of CMV, linked to the intracellular co-stimulatory domain of CD28 and the activating domain of CD3ζ. These CAR-T cells were generated by lentiviral transduction, or mRNA electroporation, inducing high CAR expression and functionality in both cases assessed by high cytokine production and antigen-induced degranulation (40). However, CMV-infected cells were resistant to killing by the CAR-T cells despite strong degranulation, which was related to the expression of anti-apoptotic factors encoded by CMV as previously found (41).

#### Hepatitis B and C

CAR-T cells might also be a good alternative for Hepatitis viral infections. Particularly noteworthy are infections caused by the hepatitis B virus (HBV), as there are currently no effective treatments for chronic cases affecting millions of people, and these infections are linked to a high mortality rate.

The literature screening found seven preclinical studies on CAR-T cells to treat HBV and a publication related to HCV infection. Most studies used scFv against HBsAg, an HBV surface protein expressed on the membrane of HBV-infected hepatocytes. HBsAg predominantly comprises the HBV small surface (S) protein with trace amounts of middle and large surface (L) proteins. Both S and L proteins are predominantly used as CAR targets. In 2008, Bohne, F. et al., showed that CARs formed by a single chain antibody fragment directed against HBV S or L protein enable primary human T cells to recognize HBsAg-positive hepatocytes, releasing cytokines and killing HBV-infected cells *in vitro* (42). After that, Krebs, K. et al., in 2013, using an *in vivo* immunocompetent mouse model, showed that HBV-specific CAR-T cells recognized HBV envelope proteins on the surface of HBV-replicating hepatocytes, engrafted and expanded *in vivo* and effectively controlled HBV replication (43). These results were later confirmed in HBV-infected human liver chimeric mice (43) and other models (44, 45).

Regarding HCV infections, only one publication was found in which CAR-T cells targeting HCV E2 glycoprotein were developed. This protein is expressed on the surface of infected cells, presenting a high mutation rate. HCV E2 CAR-T cells were found to eliminate HCV E2 glycoprotein-expressing cells and HCV-infected hepatocytes *in vitro*. Nevertheless, the significance of this study awaits confirmation through *in vivo* models that closely mimic real-life conditions (46).

#### Coronavirus

Coronaviriade is a virus family responsible for respiratory infections of a variable spectrum of severity. Three members of this family, SARS-CoV, MERS-CoV, and SARS-CoV-2 are best known because of causing severe acute respiratory disease, being the last one responsible for the Covid-19 pandemic, resulting in millions of deaths worldwide. Shortly after Covid-19 emergence, it was found that T cells were critical for controlling SARS-CoV-2 infections and might lead to long-lasting immune responses independent of mutations in antigens targeted by antibody responses. Following these observations, Keller M et al. recovered and expanded SARS-CoV-2-specific T cells from convalescent donors and manufactured them to produce IFN and TNF (47). Experimental analysis indicated that these T cells could respond to specific SARS-CoV-2 antigens, suggesting a potential benefit of using these cells in ACT for Covid-19 (47). Currently, several clinical trials are ongoing in which the efficacy of ACT to treat SARS-CoV-2 infection is being tested (48), and some CAR-T cells are being preclinically explored. Recently, a CAR-T cell directed to the SARS-CoV-2 Spike (S) receptor binding domain (RBD) has been generated and tested *in vitro* and in a mouse *in vivo* model, showing its ability to kill cells expressing RBD or full S protein (49).

More *in vitro* studies have been developed, engineering primary CD8+ T cells to express SARS-CoV-2 S protein-specific CARs, demonstrating potent cytotoxicity towards S-expressing target cells (50). NK cells and macrophages are also being evaluated in *in vitro* assays to develop CARs as other cells with high antiviral potential (51, 52). Janice Oh et al. generated TCR transgenic CD8+ T cells for other coronaviruses using TCR sequences identified in SARS-specific T cells isolated from recovering patients. Using TCR gene transfer, they genetically modified T cell lymphocytes derived from healthy individuals to target SARS, demonstrating their ability to generate cytokines and cytotoxic proteins that could help control SARS-CoV (53).

However, despite the high effector activity after stimulation with viral antigens or against viral antigen-expressing cells observed in all these studies, none of them analysed the ability of these cells to control viral replication *in vitro* or viral infection *in vivo*. Thus, the potential of CAR-T or TCR-T cells to treat infections caused by SARS-Cov/-2 is still unclear.

#### Non-viral infections

CAR cell therapies have also been developed for other severe non-viral infections. For example, invasive fungal opportunistic infections cause high morbidity and mortality in immunocompromised patients, especially those with haematological cancer. The most frequent is caused by *Aspergillus fumigatus*, a fungus that is ubiquitously present in the environment. This fungus can colonize the lung, resulting in local pulmonary damage or developing angio-invasion and haematogenous spread, especially in severe immunocompromised patients undergoing HSCT, ultimately causing invasive pulmonary aspergillosis (IPA).

In 2014, Kumaresan, P.R. et al., fashioned a glycan-specific cell receptor, Dectin-1, into a CAR by fusing the extracellular domain of human Dectin-1 to human CD28 transmembrane and cytoplasmic domains and the CD3-ζ signalling motif. CAR specificity and functionality were verified against *Aspergillus hyphae in vitro* and *in vivo*, showing high efficacy in controlling fungal growth (54). Eight years later, in 2020, the same group published another CAR T-cell against *Cryptococcus* spp infections, which infect more than 1 million people each year. In this new approach, the target of the CAR was the glucuronoxylomannan (GXM) present in *Cryptococcus* capsule. They used the same CAR structure as described above, but Dectin-1 domain was replaced by an ScFv against GXM (55).

More recently, a CAR directed to an epitope on the cell wall of *A. fumigatus* has been designed and employed to modify both CD4+ and CD8+ T cells, which were then assessed in preclinical *in vitro* and *in vivo* models of IPA. Interestingly, the results indicated that although CAR-T shows discrete direct antifungal activity, this activity was further enhanced by macrophages activated by CAR-T cell-derived cytokines, which help to control fungal infection (56). This result suggests that the efficacy of CAR-T cell therapy might be affected by patient immune status, and a combination of immunostimulatory drugs with CAR-T cells might be a good alternative to enhance efficacy in highly immunocompromised patients.

Tuberculosis, especially multidrug-resistant, or extensively drug-resistant isolated (MDR/XDR-TB) caused by the bacillus *Mycobacterium tuberculosis*, is one of the leading causes of death worldwide, with an estimated 1.3 million deaths in 2020 (57). This pathogen has developed several evolution advantages that convert it into an attractive immunotherapy target. Multiple and extended antimicrobial treatments are needed to cure it, generally resulting in poor adherence and elevated toxicity. In addition, *M. tuberculosis* can achieve a non-replicating state due to specific interactions with the host, forming granulomas that isolate bacteria from host cells, favoring antibiotic resistance. Thus, therapy failure is common in these patients, and a combination of multiple antibiotics, with the consequent increase in toxicity, is required to treat the infection. Thus, alternative therapies like CAR cells could be potentially effective against this intracellular pathogen. Although, at present, CAR cells have not been developed for this infection, different approximations have been proposed to identify antigens against which antibodies can be generated as a basis for subsequent CAR development.

One of them suggests the identification of antibodies against bacterial antigens naturally produced in TB latently infected patients that will be used to generate a scFv fragment and, subsequently, a CAR for T or NK cells (58). In addition to T and NK cells, NKT cells play an essential role in TB infections. NKT cells express receptors that recognize some lipids presented in the mycobacterial cell wall, and they have been shown to participate in the control of TB infection in several models (59), including correlational studies in humans (60–63). All these findings suggest that these cells could be used directly in ACT or their receptor used as recognizing domains in CARs. Alternatively, CAR-NKT cells could be developed for TB treatments, which might help to control Tb infection by recognizing bacteria using both their natural receptors and the CAR, overcoming potential mutations in antigens recognized by the CAR. However, all these suggestions will need to be developed and tested in proper models of TB infection.

## Discussion: realities, limitations, and future perspectives

Chronic viral infections constitute the perfect conceptual scenario for the application of CAR therapy. The majority of clinical and preclinical studies have been conducted in HIV infection, including a phase 2 clinical trial. These studies have confirmed the feasibility and safety of CAR-T therapy in this context but have not achieved sufficient power to demonstrate therapeutic efficacy, either alone or in combination with ART.

The risk of developing secondary neoplasms in hematological patients with chronic EBV latency has also brought this objective into focus for the development of immunotherapy. Despite promising results demonstrated *in vitro* and *in vivo* models, observed through tumor regression, there are still no available clinical applications, although several trials are currently recruiting. ACT, in the case of CMV infections, has marked a significant step in applying personalized immunotherapy in hematological patients. In this clinical profile, CMV infection may promote GvHD or graft dysfunction. Additionally, with the currently available antiviral treatment, achieving sustained viral clearance can be challenging, coupled with potential toxicity and the risk of developing resistance. To date, several preclinical studies utilizing CAR therapy have demonstrated effectiveness in controlling CMV viral replication in animal and laboratory models.

ACT has also been clinically tested in SARS-CoV-2 infections, where its utility is likely in onco-hematological patients with persistent Covid-19. In experimental models, the main cell types for CAR development have been T lymphocytes and NK cells, demonstrating cytokine production with antiviral activity but without achieving clinical impact due to a lack of viral load control.

Invasive infections caused by opportunistic fungi, such as *Aspergillus fumigatus* or *Cryptococcus* spp, currently represent the most significant field of research for the application of CAR therapy. The clinical characteristics of patients suffering from these infections, with a lower threshold of immunotolerance to infections, the risk of accumulated toxicity due to multiple lines of treatment, and a higher prevalence of secondary neoplasms, support the establishment of new research lines and the application of innovative therapies. CAR therapy has already demonstrated *in vitro* and in animal models the production of granzymes and cytokines with fungicidal activity. Additionally, it has been observed that these molecules induce secondary activation in the host of other cells involved in the immune response against fungi, suggesting the use of this treatment as an option for activating the natural host immune response. However, despite the results obtained in preclinical studies, no published data still endorse its use in patients.

Infections due to multidrug-resistant (MDR) bacteria cause 5 million deaths yearly. The WHO (World Health Organization) considers this issue a global public health threat, estimating an increase in their incidence in the coming years (59, 64). It makes the development of novel antimicrobials critical. However, research in this area is scarce because of the ability of bacteria to evolve quickly and generate resistance to “traditional” treatments, so it is imperative to look for alternative strategies like phage therapies, vaccines, or immunotherapy, including ACT (65). Chronic bacterial infections may be considered a promising target for CAR cells due to their similarities to the tumour immune microenvironment, the potential for toxicity associated with extended antimicrobial therapies, and the risk of developing antimicrobial resistance. However, uptodate no preclinical or clinical studies testing CAR cells in chronic bacterial infections have been developed.

The potential benefits of CAR therapy in chronic infections would primarily entail using immunotherapy tailored to the specific microorganism and individual patient. This approach would offer viable alternative treatments in the case of multi or pandrug-resistant bacteria (such as *Pseudomonas aeruginosa, Acinetobacter baumannii, Klebsiella pneumoniae* or *Mycobacterium tuberculosis*), for which current therapeutic options are limited. In these cases, it is often necessary to resort to antibiotics with questionable efficacy and high toxicity (colistin, tigecycline, cefiderocol). Therefore implementing CAR therapy emerges as a safer and more efficacious alternative. On the other hand, this therapeutic strategy can potentially abbreviate the prolonged durations of antibiotic treatments, particularly in osteoarticular or endovascular infections, where there is an accumulating risk of resistance development and poor treatment tolerance.

Concurrently, this therapeutic approach would offer new options for many infections that are now considered impossible to cure, primarily those associated with cardiac electronic devices or orthopedic prosthetic devices. These patients live with the infection, controlled through lifelong antibiotic treatment. Lastly, the integration of immunotherapy into the management of challenging infections would yield considerable population-wide benefits. It would be able to furnish therapeutic alternatives for bacteria displaying multi-drug resistance, disseminating within healthcare facilities and among patients, thereby giving rise to public health problems and elevated morbidity and mortality rates.

Not all infectious diseases are equally suitable for CAR cell therapy. For instance, fulminant or acute infections are not good candidates because CAR cell production can take about 2 weeks. Most community and hospital-acquired infections would not fit within the currently approved CAR cell paradigm because of their rapid clinical evolution and the need for immediate action. This limitation can be overcome by using CAR cells generated from allogeneic cell sources from healthy donors (i.e., NK, NKT, or γδ T cells) that could be frozen and stored to be immediately used to treat acute infection patients (66). This alternative could be, for example, used to treat acute viral infections like severe COVID-19. However, in this case, traditional autologous CAR cells could be very useful to treat long COVID-19 patients. In addition, autologous CAR cells could be used to treat prophylactically highly immunocompromised patients with a high risk of developing opportunistic and chronic infections. In this case, a precise stratification protocol should be used to include patients who will benefit most from these therapies.

Regarding chronic bacterial infections, candidates for CAR therapy include tuberculosis, non-tuberculous mycobacterial infections, osteomyelitis, and foreign-body infections that usually show a course with biofilm (prosthetic joint infections, cardiovascular devices infections, or endocarditis). Secondly, the infection should be monomicrobial to be able to select the antigen–directed CAR cell. A co–viral or bacterial infection would limit the specificity of manufactured cells, affecting the immune response, although CARs directed against two different antigens could be developed. Finally, CAR cells would probably be more effective in immunocompromised patients or those who respond weakly to viruses or vaccines, possibly providing longer-term protective immunity. In this case, and depending on the immunosuppressive characteristics, allogeneic CAR cells from healthy patients (i.e., allogeneic NK, NKT, or γδT cells that have shown an excellent safety profile) or CAR cells derived from bone marrow precursors might be necessary to treat these type of patients.

Another important limitation for increasing the field of application of CAR cells in infectious diseases is the limited number of microbial antigens. Some of them might present mutations that would overcome CAR efficacy. Thus, identifying novel microbial antigens exposed on the surface of the pathogen and/or infected cell is an emerging area of research. Here, the highly developed field of immunogenomics in oncology will be critical to decipher new targets against which CAR will be generated. This field studies the immune system and its role in disease by exploiting omics data using bioinformatic tools. In CAR immunotherapy, computational methods exist to identify surface or secreted proteins that may induce a protective response in the host (67). Various bioinformatics tools exist to predict this kind of epitopes that use peptidic sequences as input (68). For example, several bioinformatic analysis have been reported trying to identify putative antigens from *Mycobacterium ulcerans*, a bacteria that cause Buruli ulcer.

In a work by Ishwarlall et al., the authors aimed to identify antigenic CD8^+^ and CD4^+^ T-cell and B-cell epitopes from the membrane transporter proteins from *M. ulcerans (* 69). First, proteomes from *M. ulcerans* were downloaded and submitted to a database that accurately predicts potentially pathogenic proteins. Then, identified proteins were used for topology analysis, and those identified as having an outer topology were selected. These proteins were used as an input for CD4 and CD8 epitope prediction. Next, predicted no-namers were classified based on the physicochemical properties of the peptides for antigenicity analysis, selecting bacteria as a target organism. Finally, resulting antigenic no-namers were examined based on their amino acid properties and to predict peptide-MHC complexes. From a total of 9906 potentially virulent proteins identified, the screening of CD8^+^ and CD4^+^ T-cell epitopes yielded 178 CD8^+^ and 245 CD4^+^ T-cell epitopes. Following antigenicity analysis, 80 CD8^+^ and 89 CD4^+^ antigens were identified. Also, 14 antigenic B-cell epitopes were detected. Similar procedures have been used in works by Mohinani et al. and Nain et al. that aim to identify outer proteins in *M. ulcerans* susceptible to acting as antigens (70, 71). In summary, *in silico* prediction of epitopes could help identify new therapeutic targets in infectious diseases.

Another significant constraint is the cost associated with CAR production, estimated in oncology therapy to range between 370,000 and 450,000 euros. Although in-house CARs, i.e., treatments manufactured and delivered to patients within the same clinical center, could make these treatments faster and cheaper, the costs of their development and the still-unvalidated clinical efficacy hinder advances in research and limit clinical application. The goal of our review has been solely to capture the reality of its use and propose a conceptual scenario for potential applications.

Conversely, we must also consider potential adverse effects reported following CAR administration. In haematological patients, cytokine release syndrome (CRS) has an incidence reaching up to 50%. This syndrome involves an exacerbated inflammatory response due to the hyperstimulation of T cells (72), which can lead to fever and multiorgan dysfunction. Neurological toxicity, known as immune effector cell-associated neurotoxicity syndrome, has also been described in 20-70% of patients, either accompanying or not accompanying CRS (73). This can result in a wide range of symptoms, from confusion and language impairments to cerebral edema. Both conditions can be potentially fatal and require early treatment with corticosteroids and tocilizumab. Paradoxically, other relevant infusion-related complications may include prolonged cytopenias and hypogammaglobulinemia, which could be associated with the development of secondary infections, although the impact of all these adverse effects beyond current hematological indications is unknown.

Antimicrobial treatment has drastically increased life expectancy during the last 70 years. However, the increased complexity of pathogen resistance mechanisms, the (re)emergence of new pathogens, and the spread of infectious diseases due to global changes prompt the development of new therapeutic strategies. In this context, the advancement of novel immunotherapies, such as CAR cells, which have demonstrated remarkable effectiveness in cancer, should be harnessed and applied to infectious diseases. Although several preclinical studies have demonstrated the safety and efficacy of CAR treatment in different types of infections, it is necessary to progress to phase I and phase II clinical trials to ensure its application in patients.

Infectious diseases serve as a quintessential model for understanding the immune response, making them an ideal candidate for implementing these groundbreaking approaches. Ideally, immune-modified cells can be selectively directed against pathogen-derived antigens, utterly different from host antigens, a key issue for safe and efficient immunotherapy that, in several cases, limits oncology applications. The immune system returns to its origins, using the host–pathogen evolutionary conflicts to design efficient therapies to fight infection.

## Data availability statement

The original contributions presented in the study are included in the article/**Supplementary Material**. Further inquiries can be directed to the corresponding authors.

## Author contributions

ER: Writing – original draft, Writing – review & editing. CP: Investigation, Methodology, Writing – original draft. GP-S: Conceptualization, Data curation, Writing – original draft. SL-G: Formal analysis, Investigation, Methodology, Writing – original draft. LM-L: Investigation, Methodology, Writing – review & editing. SA: Conceptualization, Formal analysis, Writing – original draft. CT: Resources, Supervision, Writing – review & editing. SR: Project administration, Software, Supervision, Writing – original draft. ED-B: Formal analysis, Investigation, Supervision, Writing – original draft. MA: Methodology, Supervision, Validation, Writing – review & editing. EG: Supervision, Validation, Visualization, Writing – review & editing. RS-P: Validation, Visualization, Writing – original draft. JP: Conceptualization, Investigation, Writing – review & editing, Writing – original draft. JP-P: Supervision, Validation, Writing – review & editing. AR-L: Writing – original draft, Writing – review & editing.
