# Supplementary material for: CAR Immunotherapy for the treatment of infectious diseases: a systematic review
Source: Front Immunol. 2024 Jan 30;15:1289303. doi: 10.3389/fimmu.2024.1289303 (PMC10861799; doi:10.3389/fimmu.2024.1289303)
Supplement: Supplementary file 2 [file Table_1.docx]

**Supplementary Table 1.** Pubmed search strategy of literature about CAR cells in infectious diseases.

| 1. Search terms related to chimeric antigen receptor (N= 7,830) |
| --- |
| ("Receptors, Chimeric Antigen"[Mesh]) OR ((((((((((((Antigen Receptors, Chimeric[Title/Abstract]) OR (Artificial T-Cell Receptors[Title/ Abstract])) OR (Artificial NK-Cell Receptors[Title/ Abstract])) OR (Artificial T Cell Receptors[Title/Abstract])) OR (Artificial NK Cell Receptors[Title/Abstract])) OR (Receptors, Artificial T-Cell[Title/Abstract])) OR (Receptors, Artificial NK-Cell[Title/Abstract])) OR (T-Cell Receptors, Artificial[Title/ Abstract])) OR (NK-Cell Receptors, Artificial[Title/ Abstract])) OR (Chimeric T-Cell Receptors[Title/Abstract])) OR (Chimeric NK-Cell Receptors[Title/Abstract])) OR (Chimeric T Cell Receptors[Title/Abstract])) OR (Chimeric NK Cell Receptors[Title/Abstract])) OR (Receptors, Chimeric T-Cell[Title/Abstract])) OR (Receptors, Chimeric NK-Cell[Title/Abstract])) OR (T-Cell Receptors, Chimeric[Title/Abstract])) OR (NK-Cell Receptors, Chimeric[Title/Abstract])) OR (Macrophages-Cell Receptors, Chimeric[Title/Abstract])) OR (Chimeric Antigen Receptors[Title/Abstract])) OR (Chimeric Immunoreceptors[Title/Abstract])) OR (Immunoreceptors, Chimeric[Title/Abstract])) |
| 1. Search terms related to infection diseases (N= 725,939) |
| ("communicable diseases"[MeSH Terms] OR "bacteria diseases"[Text Word] OR "viral diseases"[Text Word] OR "fungal diseases"[Text Word] OR "bacterial infections"[Text Word] OR "viral infections"[Text Word] OR "fungal infections"[Text Word])) |
| 1. Search terms related to leukemia, myeloma and lymphoma (N=271,081) |
| ("leukemia"[Title] OR "Myeloma"[Title] OR "CD19"[Title] OR "lymphoma"[Title]) |
| 1. Combination of three research terms (N=105) (Query1 AND Query2) NOT (Query3) |
| (("Receptors, Chimeric Antigen"[Mesh]) OR ((((((((((((Antigen Receptors, Chimeric[Title/Abstract]) OR (Artificial T-Cell Receptors[Title/ Abstract])) OR (Artificial NK-Cell Receptors[Title/ Abstract])) OR (Artificial T Cell Receptors[Title/Abstract])) OR (Artificial NK Cell Receptors[Title/Abstract])) OR (Receptors, Artificial T-Cell[Title/Abstract])) OR (Receptors, Artificial NK-Cell[Title/Abstract])) OR (T-Cell Receptors, Artificial[Title/ Abstract])) OR (NK-Cell Receptors, Artificial[Title/ Abstract])) OR (Chimeric T-Cell Receptors[Title/Abstract])) OR (Chimeric NK-Cell Receptors[Title/Abstract])) OR (Chimeric T Cell Receptors[Title/Abstract])) OR (Chimeric NK Cell Receptors[Title/Abstract])) OR (Receptors, Chimeric T-Cell[Title/Abstract])) OR (Receptors, Chimeric NK-Cell[Title/Abstract])) OR (T-Cell Receptors, Chimeric[Title/Abstract])) OR (NK-Cell Receptors, Chimeric[Title/Abstract])) OR (Macrophages-Cell Receptors, Chimeric[Title/Abstract])) OR (Chimeric Antigen Receptors[Title/Abstract])) OR (Chimeric Immunoreceptors[Title/Abstract])) OR (Immunoreceptors, Chimeric[Title/Abstract]))) AND ("communicable diseases"[MeSH Terms] OR "bacteria diseases"[Text Word] OR "viral diseases"[Text Word] OR "fungal diseases"[Text Word] OR "bacterial infections"[Text Word] OR "viral infections"[Text Word] OR "fungal infections"[Text Word]))) NOT ("leukemia"[Title] OR "Myeloma"[Title] OR "CD19"[Title] OR "lymphoma"[Title]) |

**Supplementary Table 2**. Scopus search strategy of literatures about chimeric antigen receptor cell in infection diseases.

| 1. **Search terms related to chimeric antigen receptor (N=**10,648 **)** |
| --- |
| ( TITLE ( car ) OR KEY ( car ) OR TITLE-ABS-KEY (“chimeric antigen receptor”) ) AND ( TITLE-ABS-KEY (“T cell”) OR TITLE-ABS-KEY (“NK cell”) ) AND ( LIMIT-TO ( DOCTYPE, “re”) OR LIMIT-TO ( DOCTYPE, “ar”) ) |
| 1. **Search terms related to infection diseases (N=**528,427**)** |
| ( ( TITLE ( infection ) OR KEY ( infection ) ) OR TITLE-ABS-KEY ( infect* PRE/0 disease ) ) AND ( TITLE-ABS-KEY ( bacteri* ) OR TITLE-ABS-KEY ( viral ) OR TITLE-ABS-KEY ( virus* ) OR TITLE-ABS-KEY ( fung* ) ) AND PUBYEAR > 2012 AND ( LIMIT-TO ( DOCTYPE, “re”) OR LIMIT-TO ( DOCTYPE, “ar”) ) |
| 1. **Search terms related to leukemia, myeloma and lymphoma (N=**559,659 **)** |
| ( TITLE ( leukemia* )  OR  TITLE ( lymphoma* )  OR  TITLE ( tumo* )  OR  ( cytokine  AND  release ) )  AND  PUBYEAR  >  2012  AND  ( LIMIT-TO ( DOCTYPE ,  “re” )  OR  LIMIT-TO ( DOCTYPE ,  “ar” ) ) |
| **Combination of three research terms (N=**260**) (**Query1 AND Query2**) NOT** (Query3**)** |
| ( TITLE ( car )  OR  KEY ( car )  OR  TITLE-ABS-KEY ( “chimeric antigen receptor” ) )  AND  ( TITLE-ABS-KEY ( “T cell” )  OR  TITLE-ABS-KEY ( “NK cell” ) )  AND  ( ( TITLE ( infection )  OR  KEY ( infection ) )  OR  TITLE-ABS-KEY ( infect*  PRE/0  disease ) )  AND  ( TITLE-ABS-KEY ( bacteri* )  OR  TITLE-ABS-KEY ( viral )  OR  TITLE-ABS-KEY ( virus* )  OR  TITLE-ABS-KEY ( fung* ) )  AND NOT  ( TITLE ( leukemia* )  OR  TITLE ( lymphoma* )  OR  TITLE ( tumo* )  OR  ( cytokine  AND  release ) )  AND  ( LIMIT-tO ( DOCTYPE ,  “re” )  OR  LIMIT-TO ( DOCTYPE ,  “ar” ) ) |

**Supplementary Table 3.** Cochrane search strategy of literatures about chimeric antigen receptor T cell in infection diseases.

| **Search terms related to chimeric antigen receptor cell and infection diseases (N=**86 **)** |
| --- |
| ((CAR NK cell):ti,ab,kw OR (CAR T cell):ti,ab,kw OR (CAR-T Therapy):ti,ab,kw OR (CAR-NK Therapy):ti,ab,kw OR (chimeric antigen receptor):ti,ab,kw OR (CAR T immunotherapy):ti,ab,kw OR (engineered t cell):ti,ab,kw) AND (INFECTION*):ti,ab,kw |
